# Exploring autism symptoms in an Australian cohort of patients with Prader-Willi and Angelman syndromes

**DOI:** 10.1186/s11689-018-9242-0

**Published:** 2018-08-06

**Authors:** Emma K. Baker, David E. Godler, Minh Bui, Chriselle Hickerton, Carolyn Rogers, Mike Field, David J. Amor, Lesley Bretherton

**Affiliations:** 1 Cyto-Molecular Diagnostic Research Laboratory, Victorian Clinical Genetics Services and Murdoch Children’s Research Institute, Royal Children’s Hospital, Parkville, VIC Australia; 2 Department of Paediatrics, Faculty of Medicine, Dentistry and Health Sciences, University of Melbourne, Parkville, VIC Australia; 3 Centre for Epidemiology and Biostatistics, Melbourne School of Population and Global Health, University of Melbourne, Parkville, VIC Australia; 4 Genetics Education and Health Research, Murdoch Children’s Research Institute, Royal Children’s Hospital, Parkville, VIC Australia; 5 Genetics of Learning Disability Service (GOLD Service), Hunter Genetics, Newcastle, NSW Australia; 6 Neurodisability and Rehabilitation, Murdoch Children’s Research Institute, Royal Children’s Hospital, Parkville, VIC Australia; 7 Child Neuropsychology, Murdoch Children’s Research Institute, Royal Children’s Hospital, Parkville, VIC Australia; 8 Melbourne School of Psychological Sciences, University of Melbourne, Parkville, VIC Australia

**Keywords:** Prader-Willi syndrome, Angelman syndrome, Autism, ADOS, IQ

## Abstract

**Background:**

Prader-Willi syndrome (PWS) and Angelman syndrome (AS) are neurodevelopmental disorders that are caused by abnormal expression of imprinted genes in the 15q11-13 region. Dysregulation of genes located in this region has been proposed as a susceptibility factor for autism spectrum disorder (ASD) in both disorders.

**Methods:**

This study aimed to explore symptoms of ASD in 25 PWS and 19 AS individuals aged between 1 and 39 years via objective assessment. Participants completed the Autism Diagnostic Observation Schedule-2nd Edition (ADOS-2) and a developmentally or age-appropriate intellectual functioning assessment. All participants had their genetic diagnosis confirmed using DNA methylation analysis and microarray testing of copy number changes within the 15q11-13 region.

**Results:**

Participants with PWS had significantly higher overall and social affect calibrated severity scores (CSS) on the ADOS-2 compared to AS participants (*p* = .0055 and .0015, respectively), but the two groups did not differ significantly on CSS for the repetitive and restricted behaviour domain.

**Conclusions:**

PWS cases presented with greater symptoms associated with ASD compared to individuals with AS. Mental health issues associated with PWS may contribute to elevated symptoms of ASD, particularly in adolescents and adults with PWS.

**Electronic supplementary material:**

The online version of this article (10.1186/s11689-018-9242-0) contains supplementary material, which is available to authorized users.

## Background

Prader-Willi syndrome (PWS) and Angelman syndrome (AS) are neurodevelopmental disorders associated with deficits in intellectual functioning. Both disorders are caused by opposing effects of copy number or DNA methylation changes at the same locus on chromosome 15 [1]. PWS results from the loss of function of paternal genes from chromosome 15q11.2-q13 while AS from the absence of function of the maternal genes in the same region [2]. In PWS, three primary mechanisms result in the lack of expression of key genes and thus the resulting condition: (i) a deletion from the paternally contributed chromosome 15 (65–75% of cases); (ii) maternal uniparental disomy (matUPD; 20–30% of cases) and (iii) an imprinting centre defect (ICD; 1–3% of cases). Similarly, deletions from the maternally contributed chromosome 15 are the most common cause of AS (~ 75% of cases). Paternal UPD (patUPD) occurs in approximately 1–2% of AS cases and ICD in approximately 1–3% of cases [2]. Approximately 5–10% of AS cases result from a mutation in the ubiquitin-protein ligase E3A gene (*UBE3A*) [2]. In these cases, no deletion, UPD or methylation abnormality is observed and can be familial [2]. The remaining ~ 10% of cases have the disorder as a result of an as-yet unidentified genetic mechanism that is not detected by standard diagnostic testing [3].

General intellectual functioning in PWS ranges from average abilities to moderate intellectual disability (ID), with the majority of individuals with PWS falling in the mild ID range (IQ between 55 and 70) [4]. AS is commonly associated with severe intellectual impairment: most individuals with AS are non-verbal, though some individuals may have a few single words and in very rare cases some individuals are able to use phrase speech [5]. However, receptive language abilities are significantly better developed than expressive language abilities [6]. Individuals with AS are reported to plateau at a developmental level usually attained by typically developing infants of approximately 24–30 months [7]. Thus, the use of standardised, age-appropriate, assessments is difficult, and valid IQ scores are rarely obtained.

Children with PWS are frequently reported to have social difficulties that lie along the same continuum of autism spectrum disorder (ASD), though the severity is reported not to be as great as those diagnosed with ASD [8]. In contrast, individuals with AS are usually eager to communicate and engage socially with others [9]. However, repetitive and restricted behaviours (RRBs) are commonly reported in both those with PWS and AS [10]. In particular, those with PWS are reported to order and arrange objects and have insistence on routines, as well as repetitive speech [11], while hand and body mannerisms (e.g. hand flapping) are reported in nearly all cases of those with AS [12].

Overexpression of maternally expressed genes in the 15q11-13 region, including *UBE3A*, has been proposed to be a susceptibility factor for ASD [13]. Thus, higher rates of ASD might be expected in those PWS patients with the matUPD subtype compared to deletion cases and those with AS [14]. The most comprehensive PWS study to date assessed the prevalence of ASD in 146 children and adolescents aged 4–21 years [15]. Using the ADOS-2, 21.9% of the samples were classified as having ASD. Those individuals meeting criteria for ASD on the ADOS-2 were subsequently reviewed by an expert clinical team, which reduced the rate of ASD to 12.3% (18 children and adolescents). Individuals with the matUPD sub-type were more likely to meet criteria for ASD compared to the other sub-types (14 matUPD versus 2 ICD and 2 deletions). Interestingly, 46% of individuals with PWS had difficulties with social reciprocity items on the ADOS-2 (e.g. amount of reciprocal communication, quality of social overtures, social response and rapport) despite being below the autism-spectrum threshold. ADOS-2 overall and social affect (SA) calibrated severity scores (CSS) were negatively correlated with verbal, non-verbal and composite IQ scores derived from the Kaufman Brief Intelligence Test-2nd Edition (KBIT-2) [16].

Peters and colleagues [12] assessed 19 children with AS using the Autism Diagnostic Observation Schedule-Generic (ADOS-G) [17] in addition to independent evaluation of autistic symptoms by a team of clinicians using DSM-IV [18] criteria. Based on the DSM-IV, 42% of the sample met criteria for ASD and all these children also met criteria for ASD on the ADOS-G. Those AS children who did not receive an ASD diagnosis nonetheless exhibited characteristics of ASD, primarily stereotyped hand and body mannerisms. In describing the AS individuals who met criteria for ASD, the authors noted that they rarely directed vocalisations, were not responsive to their name being called and did not exhibit shared enjoyment in interactions with others. Generally, these children were more focused on objects and the repetitive use of these objects and made few social overtures to others. In addition, those children meeting criteria for ASD had significantly lower mental scores on the Bayley Scales of Infant Development [19] compared to their non-ASD counterparts.

Overall autism symptoms appear to be highly prevalent in individuals with PWS and AS. Until recently, the majority of research had used autism screeners rather than objective assessment to determine prevalence rates of ASD in these conditions. In addition, there is limited research comparing the overlap of symptoms between those with PWS and AS. Considering that both disorders are caused by opposing effects on imprinting resulting in abnormal expression of the same gene cluster on chromosome 15, exploring the similarities and differences in these two groups, has the potential to shed light on the genetic susceptibility factors associated with ASD more generally and, more specifically, to improve understanding of the natural history of both PWS and AS. Further evidence supporting an association between the PWS/AS locus and autism comes from the observation that autism is common in individuals who have a duplication on the maternally inherited copy of 15q11-13 [20]. In particular, increased gene dosage of *UBE3A* has been shown to result in defective social interaction and communication and increased repetitive behaviours in mice [21]. Moreover, identifying specific symptoms associated with the presence of ASD in these populations can assist diagnosticians in identifying ASD in these patients. By detecting ASD in these cases, autism-specific interventions can be employed to assist in the treatment of ASD symptoms that may exacerbate core symptoms of the genetic diagnosis.

This study aimed to explore, using objective assessment, symptoms associated with ASD in well-characterised cohorts of Australian individuals with PWS and AS, and to compare symptom profiles between the two disorders. It was hypothesised that those with PWS would have significantly greater number and severity of symptoms associated with ASD compared to individuals with AS.

## Methods

### Participants

Twenty-five individuals with PWS (44.0% male) and 19 individuals with AS (52.6% male) participated in the study. PWS participants were aged between 1.51 and 32.24 years (median (Md) = 7.15, interquartile range (IQR) = 8.78), while AS participants were aged between 2.76 and 39.74 years (Md = 6.93, IQR = 9.32). The two groups did not differ significantly on sex (*p* = .761) or age (*p* = .934). Table 1 presents further demographic information for the two groups.

**Table 1:** Demographic information for the PWS and AS groups

	PWS	AS
Medication use		
Growth hormone	68.0%	0%
Anticonvulsant	8.0%	73.7%
Genetic subtype		
Deletion	24.0%	47.4%
UPD	64.0%	15.8%
Imprinting centre defect	8.0%	0%
Abnormal methylation^a^	4.0%	5.2%
*UBE3A* mutation	NA	31.6%

^a^Methylation analysis confirmed PWS or AS diagnosis, but further analysis to confirm specific subtypes had not been undertaken

#### Recruitment

Participants were recruited through Victorian Clinical Genetics Services (VCGS); Hunter Genetics; the PWS Clinic at the Royal Children’s Hospital, Melbourne; the Prader-Willi Association of Australia; and the Foundation for Angelman Syndrome Therapeutics (FAST). To be included in the study, verbal participants were required to speak English and non-verbal participants were required to be exposed to English within the home. Original genetic diagnostic reports were retrieved to confirm diagnoses. All participants had their genetic diagnosis confirmed using DNA methylation analysis and microarray testing for copy number changes within the 15q11-13 region. Standard diagnostic protocols from clinical diagnostic laboratories were used for molecular diagnosis. Individuals were excluded from the study if they had the following: any other genetic condition of known clinical significance, any other medical or neurological conditions (e.g. severe head trauma, stroke) and/or inadequately controlled seizures.

Interested families returned an expression of interest form to the research team and were subsequently contacted to determine eligibility. Families that were eligible for the study attended an appointment to undertake the assessments.

### Measures

#### Intellectual functioning

The Mullen Scales of Early Learning (MSEL) [22] was used to assess intellectual functioning in PWS children aged 12 months to 2 years and 11 months and all individuals with AS. The MSEL is a standardised cognitive assessment for infants through to early childhood (birth to 70 months), specifically assessing the domains of Visual Reception, Fine Motor, Receptive Language and Expressive Language. Thus, the MSEL was used beyond its normative age range in some individuals with AS. Given the apparent developmental plateau in children with AS at approximately 24–30 months [7], it is more appropriate to assess them with a measure that is developmentally appropriate. As the MSEL does not provide separate VIQ and NVIQ scores this was calculated for each participant. Derivative IQ scores were calculated to obtain estimates of verbal IQ (VIQ) and non-verbal IQ (NVIQ) for those children who obtained a *T*-score of at least 20 on each sub-scale. Derivative VIQ scores are calculated by adding the two *T*-scores from the two verbal sub-scales and then doubling this sum. The Early Learning Composite (ELC) conversion table is then used to determine the derivative VIQ. Derivative NVIQ scores are calculated by using the two non-verbal subscales. The Early Learning Composite (ELC) was used as a proxy for Full Scale IQ (FSIQ) [23] in these participants. Ratio IQ scores were calculated to obtain estimates of VIQ, NVIQ and FSIQ in those individuals who obtained *T*-scores < 20 and those AS participants who were aged > 70 months. Briefly, ratio VIQ scores are calculated by averaging the age equivalents of the two VIQ subscales and then dividing this average by the individual’s chronological age and multiplying by 100. The same procedure is used to calculate ratio NVIQ scores using the two age equivalent scores of the nonverbal subscales. FSIQ scores are calculated by using both verbal and nonverbal subscale age equivalents (see Richler et al. [24] for a full description of the methods).

Children with PWS aged between 3 and 6 years and 11 months were assessed with the Wechsler Preschool and Primary Scale of Intelligence-3rd Edition (WPPSI-III) [25]. Those aged between 7 and 16 years and 11 months were assessed with the Wechsler Intelligence Scale for Children-4th Edition (WISC-IV) [26], and those aged 17 years and older were assessed with the Wechsler Adult Intelligence Scale-4th Edition (WAIS-IV) [27].

#### Autism spectrum disorder symptoms

The Autism Diagnostic Observation Schedule-2nd Edition (ADOS-2) [28] was used to assess autism symptoms in both groups of participants. The ADOS-2 is a semi-structured assessment conducted by an adult who is unfamiliar to the participant. There are five modules which are used depending on the age and language abilities of the person to be assessed. For all modules, calibrated severity scores (CSS) can be derived for the total score as well as the social affect (SA) and restricted and repetitive behaviours (RRB) domains [29, 30] which allows comparison of the severity of autism symptoms across the five modules. The ADOS-2 was completed by the first author who has demonstrated > 80% coding reliability across all five modules.

### Data analysis

Non-parametric Mann-Whitney *U* test was used to compare the difference between groups. To compare the difference in proportions between groups, Fisher’s exact test was used. Summary statistics were then presented as median (Md), interquartile range (IQR) or proportion. The two groups were compared on the key diagnostic items from the ADOS-2. Codes of 1, 2 and 3 were combined to indicate an atypical presentation on the specific behaviour while scores of 0 indicated typical presentation. False discovery rate (FDR) was applied to adjust for multiple comparisons. *p* value < 0.05 was considered as significant. Effect sizes (e.g. *r* and Φ) are included to assist in the interpretation of findings: small effects = 0.10, medium effects = 0.30 and large effects = 0.50. All analyses were conducted using statistical package SPSS (Version 24).

## Results

### Intellectual functioning

Eighteen participants with AS were assessed with the MSEL; one child with AS did not complete the MSEL. All AS children who were within the appropriate age range of the MSEL did not obtain valid scores. Thus, ratio-IQs were calculated for all AS participants. Three participants with PWS (< 3 years) were assessed with the MSEL. These three individuals obtained valid *T-*scores, and thus, derivative IQ scores were calculated for VIQ and NVIQ. Two PWS participants did not obtain valid scores on their respective assessments (WAIS-IV and WPPSI-III), and minimum scores were entered for these individuals. As expected, the PWS group had significantly higher intellectual functioning scores compared to the AS group (see Table 2). Age equivalent descriptive statistics for the four MSEL domains for AS participants are provided in the Additional file 1: Table S1.

**Table 2:** Comparison of intellectual functioning and ADOS-2 CSS variables between PWS and AS participants using Mann-Whitney *U* tests

	PWS		AS			
	*n*	Md (IQR)	*n*	Md (IQR)	*p*	*r*
Intellectual functioning						
VIQ	25	67.0 (16.5)	18	18.5 (19.5)	*2.9 × 10* ^*−6*^	.85
NVIQ	25	61.0 (18.5)	18	21.5 (21.25)	*3.0 × 10* ^*−6*^	.85
FSIQ	25	62.0 (20.0)	18	20.0 (19.75)	*3.0 × 10* ^*−6*^	.84
ADOS-2						
Overall	25	6.0 (5.5)	19	3.0 (3.0)	*.0055*	.42
SA	25	5.0 (4.5)	19	3.0 (2.0)	*.0015*	.48
RRB	25	7.0 (3.5)	19	6.0 (1.0)	.4158	.12

*p* values (*p*) in italics remain < 0.05 after adjusted for multiple testing using FDR

*n* sample size, *Md* median, *IQR* interquartile, *r* effect size

### Autism symptoms

The overall and SA CSS were significantly higher in PWS than in AS participants; however, the two groups did not differ on the RRB CSS (see Table 2). These differences between the two groups remained significant after controlling for FSIQ using multiple regression (overall CSS: *p* = .0002; SA CSS: *p* = .0004). When comparing PWS and AS participants on key diagnostic items of the ADOS-2, several differences emerged (Table 3). In particular, a significantly greater proportion of PWS participants engaged in sensory-seeking behaviours. In contrast, AS participants were more likely to display hand mannerisms compared to PWS participants. Trends (non-significant) were also observed, with medium effect sizes for pointing (more atypical in AS group) and shared enjoyment (more atypical in PWS group).

**Table 3:** Comparison of PWS and AS participants on ADOS-2 algorithm items using Fisher’s exact test

	PWS		AS			
	*n*	% atypical	*n*	% atypical	*p*	Φ
SA						
Pointing	12	33.3	19	68.5	.075	.343
Gestures	25	48.0	19	52.6	.999	.046
Eye contact	25	64.0	19	52.6	.542	.115
Facial expressions	25	64.0	19	84.2	.181	.225
Shared enjoyment	25	68.0	19	31.6	.032	.361
Showing	12	33.3	19	42.1	.717	.088
Response to joint attention	12	8.3	19	26.3	.363	.222
Initiation of joint attention	12	16.7	19	42.1	.240	.265
Quality of social overtures	25	64.0	19	78.9	.335	.162
Amount of social overtures	25	36.0	19	57.9	.223	.218
Rapport	25	84.0	19	73.7	.467	.127
Reporting^a^	13	69.2	–	–	–	–
Conversation^b^	20	75.0	–	–	–	–
Amount of communication^b^	20	35.0	–	–	–	–
Insight^a^	13	92.3	–	–	–	–
Communication of own affect^c^	5	100	–	–	–	–
RRB						
Sensory	25	40.0	19	5.3	*.013*	.397
Mannerisms	25	36.0	19	89.5	*.001*	.539
Repetitive and stereotyped behaviours	25	64.0	19	68.4	.999	.046
Stereotyped language^b^	25	56.0	–	–	–	–

*p* value (*p*) in italics remains < 0.05 after adjustment for multiple comparisons using FDR

*n* sample size, Φ effect size

^a^Module 3 and 4 item

^b^Module 2–4 item

^c^Module 4 item

Significant negative correlations were observed between all ADOS-2 CSS scores and VIQ in the PWS group. In addition, overall and SA CSS scores were associated with FSIQ in the PWS group. No significant correlations were observed between ADOS-2 CSS scores and intellectual functioning variables in the AS group (see Table 4).

**Table 4:** Spearman’s rank correlation (*ρ*) between intellectual functioning and ADOS-2 CSS for the PWS and AS groups

		VIQ		PIQ		FSIQ	
Variable	Group	*ρ*	*p* value	*ρ*	*p* value	*ρ*	*p* value
Overall CSS	PWS (*n* = 25)	− 0.57	**.** *003*	− 0.28	.169	− 0.45	*.023*
	AS (*n* = 18)	− 0.06	.817	− 0.21	.407	− 0.16	.539
SA CSS	PWS (*n* = 25)	− 0.51	*.009*	− 0.27	.193	− 0.45	*.023*
	AS (*n* = 18)	− 0.06	.799	− 0.21	.411	− 0.16	.538
RRB CSS	PWS (*n* = 25)	− 0.48	*.016*	− 0.15	.483	− 0.26	.216
	AS (*n* = 18)	− 0.06	.806	− 0.11	.670	− 0.07	.768

Those italicized *p* values remain < 0.05 after adjusting for multiple testing using FDR (notice that the *p* values in the table are raw *p* values)

## Discussion

To our knowledge, this is the first study to investigate and compare autism symptoms and intellectual functioning in well-characterised cohorts of individuals with PWS and AS using objective assessments. PWS participants had significantly higher overall and SA CSS compared to participants with AS. Individuals with AS are typically reported to be motivated to engage socially with others [31], while those with PWS are more frequently described to have social communication difficulties [32]. Although no significant group differences were observed for key diagnostic social affect items, medium effect sizes were observed for shared enjoyment and pointing. A greater proportion of individuals with PWS presented with reduced shared enjoyment with the researcher, while AS cases presented with more atypical pointing. It is likely that those with PWS present with more global social communication difficulties, increasing SA scores overall, while those with AS may present with difficulties on particular items due to other comorbidities associated with AS. In particular, motor deficits associated with AS may impair the individual’s ability to point, gesture and show items to others. Moreover, for the items pointing and showing, these need to be paired with eye contact to obtain a ‘typical’ score. The coordination of these behaviours may be difficult in those with AS, leading to a more atypical presentation. Nonetheless, these findings are similar to those of Trillinsgaard and Ostergaard [6] in which 10 children with AS + ASD (by ADOS-G) and eight children with ASD only were shown to have significant deficits on tasks requiring triadic exchanges (e.g. pointing and showing). The authors stated that such behaviours were practically non-existent in the two groups.

It has been suggested that during childhood, other developmental difficulties and behaviours associated with PWS may overshadow ASD symptoms, which may not become fully apparent until the child is older [33]. Indeed, internalising symptoms such as anxiety, low self-worth and sadness have been shown to increase with age in patients with PWS [34]. As previously reported by Molloy and colleagues [35], other developmental and behavioural problems may inflate ADOS scores, which may be the case in the participants observed here; mental health issues may be inflating scores on the ADOS in PWS patients, particularly SA scores in adolescents and adults.

The most commonly reported symptoms associated with ASD in both PWS and AS are captured by the RRB domain, which likely explains the lack of significant differences between the two groups on the RRB CSS. Nonetheless, differences emerged on the specific behaviours that are associated with this domain. In particular, PWS participants were more likely to engage in sensory-seeking behaviours compared to AS participants, while AS patients were more likely to be observed to use hand mannerisms. Hand flapping is a characteristic of individuals with AS; thus, it is not surprising that the groups differed on this item; however, sensory exploration of objects is not commonly reported for individuals with PWS. The most frequent sensory-seeking behaviours observed during the assessments included feeling textural elements of objects (*n* = 7), followed by smelling items (*n* = 5). Four children with PWS were also observed to visually inspect objects. In regard to the code of unusually repetitive interests or stereotyped behaviours, the two groups did not differ in the proportion of individuals who coded as atypical. Ten children in the AS group and eight children in the PWS group engaged in repetitive play with objects. One adult with PWS became fixated on the miniature chocolate bar during the Joint Interactive Play task. This same adult and another child engaged in repetitive food-seeking behaviour during the assessment. Seven PWS participants also discussed highly specific topics of interest. Of these seven, one participant repeatedly brought up the topic of food. Thus, some of the behaviours observed in individuals with PWS may be attributed to their PWS diagnosis, specifically food-seeking behaviour, rather than the presence of ASD.

Consistent with previous literature [15], there were significant correlations between VIQ and each of the ADOS CSS in the PWS group, whereby poorer verbal skills were associated with higher ADOS scores. Similarly, lower FSIQ scores were associated with greater overall and social affect CSS in the PWS group. However, no significant associations between ADOS CSS and intellectual functioning scores were observed in the AS group. Thus, poorer cognitive ability, particularly verbal ability, appears to be associated with symptoms of ASD in individuals with PWS.

According to Barbaro and Dissanayake [36] ‘red flags’ for ASD in young children include a lack of eye contact, interest and pleasure in others, emotional expression, directed vocalisations, joint attention skills (e.g. pointing to share interest, following other’s pointing and monitoring of others’ gaze), requesting behaviours and gestures. While in adolescents and adults, ASD may be suspected when there are persistent difficulties with social interaction, social communication and/or the presence of stereotypic behaviours. These should be accompanied by difficulties in obtaining and sustaining education or employment, difficulties in initiating and maintaining social relationships and/or previous contact with mental health or learning disability services [37]. More than 50% of individuals in each group had inconsistent eye contact and a limited range in facial expressions; however, joint attention skills (initiation and response) generally had low atypical rates in both the PWS and AS groups. Approximately 50% of individuals in each group used gestures to facilitate their communication. Thus, while some ‘red flag’ behaviours associated with ASD appear to be present in the majority of individuals with these conditions, other behaviours tend to present more typically. Thus, it is likely that behaviours such as poor eye contact and a reduction in the range of emotional expression are associated with the genetic conditions themselves, or the comorbidities associated with the conditions. However, when deficits with joint attention skills and gesturing are also observed, further assessment for ASD may be warranted.

### Limitations

One limitation of this study is the use of the ADOS in isolation and in those with a mental age below 12 months; the use of the ADOS has been cautioned in those with a mental age below 12 months [38]. Conversely, a recent paper [39] comparing diagnostic rates of ASD in children with low mental age, autistic disorder and PDD-NOS showed high stability of ASD at 2 years follow-up, prompting the authors to recommend diagnosis of ASD in these children. In our AS sample, we only had one child who had age-equivalent scores on the visual reception, fine motor and receptive language domains that were all below 12 months. Nonetheless, the AS group included in the current study had a wide age range including adults aged up to 35 years. Taking this into consideration and given the other comorbidities associated with both the AS (e.g. motor deficits) and PWS (e.g. mood disorders) groups, interpretation of ADOS scores in isolation should be cautioned. As per standard diagnostic protocols for individuals with ASD, a multidisciplinary assessment including a developmental history should be undertaken when assessing for ASD in these conditions.

Another potential limitation of the current study is the over representation of matUPD cases in the PWS cohort in comparison to typical prevalence rates. Recent research has indicated that with increasing maternal age, the prevalence of matUPD cases has also increased [40, 41]. In the current study, 66.7% of mothers with a child with matUPD were aged between 36 and 46 years at the time their child was born, compared to 40% of mothers with children with the other PWS subtypes. In addition, the ascertainment bias of the current study may have resulted in parents of children with what is deemed to be a less prevalent sub-type being more inclined to participate in research. Similarly, those individuals included in the current study may have a more severe clinical presentation than other studies that are based on population estimates. Parents of individuals with a more severe presentation may be more inclined to be involved in the study to gain more information regarding their child. Lastly, the age ranges in each cohort span several developmental stages which may impact on the results presented here. In particular, the presence of mood disorders that may manifest during adolescence and adulthood may dampen affect leading to increased social affect scores, particularly in the PWS cases.

## Conclusions

This is the first study to explore and compare objective assessments of ASD symptoms and intellectual functioning in patients with PWS and AS. Despite small sample size being a limitation, this is one of the largest studies to date of its kind in these rare disorders. While particular behaviours that are associated with an ASD diagnosis (e.g. poor eye contact, limited range of emotional expression, poor quality social overtures) were particularly prevalent in both PWS and AS individuals, joint attention skills were less commonly deficient. Differential diagnosis of ASD versus other mental health issues in PWS is particularly pertinent, as this will have implications for treatments and interventions. In addition, consideration of the common comorbidities associated with each of the conditions (e.g. motor impairments) should be taken into consideration when assessing for the presence of ASD.

## Additional file

Additional file 1: **Table S1.** Age-equivalent (months) descriptive statistics on the MSEL subscales for AS participants. (DOCX 16 kb)

## Abbreviations

ADOS-2: Autism Diagnostic Observation Schedule-2nd Edition
ADOS-G: Autism Diagnostic Observation Schedule-Generic
AS: Angelman syndrome
ASD: Autism spectrum disorder
CSS: Calibrated severity scores
FSIQ: Full Scale IQ
MSEL: Mullen Scales of Early Learning
NVIQ: Non-verbal IQ
PWS: Prader-Willi syndrome
VIQ: Verbal IQ
WAIS-IV: Wechsler Adult Intelligence Scale-4th Edition
WISC-IV: Wechsler Intelligence Scale for Children-4th Edition
WPPSI-III: Wechsler Preschool and Primary Scale of Intelligence-3rd Edition
