# Supplementary material for: Exploring autism symptoms in an Australian cohort of patients with Prader-Willi and Angelman syndromes
Source: J Neurodev Disord. 2018 Aug 6;10:24. doi: 10.1186/s11689-018-9242-0 (PMC6091196; doi:10.1186/s11689-018-9242-0)
Supplement: Supplementary file 1 — Table S1. Age-equivalent (months) descriptive statistics on the MSEL subscales for AS participants. (DOCX 16 kb) [file 11689_2018_9242_MOESM1_ESM.docx]

|  | Visual Reception | Fine Motor | Receptive Language | Expressive Language |
| --- | --- | --- | --- | --- |
| *n* | 18 | 18 | 18 | 18 |
| Mean | 20.17 | 20.11 | 23.28 | 11.06 |
| Standard Deviation | 7.42 | 6.13 | 7.56 | 4.44 |
| Median | 21.0 | 19.0 | 27.0 | 12.5 |
| Minimum | 7.0 | 7.0 | 8.0 | 3.0 |
| Maximum | 34.0 | 29.0 | 34.0 | 16.0 |

Table S1 Age Equivalent (Months) Descriptive Statistics on the MSEL Subscales for AS Participants
